# Tumor Microenvironment Modifications Recorded With IVIM Perfusion Analysis and DCE-MRI After Neoadjuvant Radiotherapy: A Preclinical Study

**DOI:** 10.3389/fonc.2021.784437

**Published:** 2021-12-21

**Authors:** François Lallemand, Natacha Leroi, Silvia Blacher, Mohamed Ali Bahri, Evelyne Balteau, Philippe Coucke, Agnès Noël, Alain Plenevaux, Philippe Martinive

**Affiliations:** ^1^ Department of Radiotherapy-Oncology, Centre Hospitalier Universitaire (CHU) de Liège, University of Liège (ULg), Liège, Belgium; ^2^ Laboratory of Tumor and Development Biology, University of Liège (ULg), Liège, Belgium; ^3^ GIGA-Cyclotron Research Centre-in vivo Imaging, University of Liège, Liège, Belgium; ^4^ Department of Radiotherapy-Oncology, Institut Jules Bordet, Université Libre de Bruxelles (ULB), Brussels, Belgium

**Keywords:** radiotherapy, surgery, cancer, neoadjuvant, DW-MRI, DCE MRI

## Abstract

**Purpose:**

Neoadjuvant radiotherapy (NeoRT) improves tumor local control and facilitates tumor resection in many cancers. Some clinical studies demonstrated that both timing of surgery and RT schedule influence tumor dissemination, and subsequently patient overall survival. Previously, we developed a pre-clinical model demonstrating the impact of NeoRT schedule and timing of surgery on metastatic spreading. We report on the impact of NeoRT on tumor microenvironment by MRI.

**Methods:**

According to our NeoRT model, MDA-MB 231 cells were implanted in the flank of SCID mice. Tumors were locally irradiated (PXI X-Rad SmART) with 2x5Gy and then surgically removed at different time points after RT. Diffusion-weighted (DW) and Dynamic contrast enhancement (DCE) MRI images were acquired before RT and every 2 days between RT and surgery. IntraVoxel Incoherent Motion (IVIM) analysis was used to obtain information on intravascular diffusion, related to perfusion (F: perfusion factor) and subsequently tumor vessels perfusion. For DCE-MRI, we performed semi-quantitative analyses.

**Results:**

With this experimental model, a significant and transient increase of the perfusion factor F [50% of the basal value (n=16, p<0.005)] was observed on day 6 after irradiation as well as a significant increase of the WashinSlope with DCE-MRI at day 6 (n=13, p<0.05). Using immunohistochemistry, a significant increase of perfused vessels was highlighted, corresponding to the increase of perfusion in MRI at this same time point. Moreover, Tumor surgical resection during this peak of vascularization results in an increase of metastasis burden (n=10, p<0.05).

**Conclusion:**

Significant differences in perfusion-related parameters (F and WashinSlope) were observed on day 6 in a neoadjuvant radiotherapy model using SCID mice. These modifications are correlated with an increase of perfused vessels in histological analysis and also with an increase of metastasis spreading after the surgical procedure. This experimental observation could potentially result in a way to personalize treatment, by modulating the time of surgery guided on MRI functional data, especially tumor perfusion.

## Introduction

Radiotherapy (RT) is commonly used in at least 50% of cancer treatments. The effects of RT are not restricted to a direct effect on tumor cells; RT also impacts the tumor microenvironment (TME) (1, 2). The role of the TME in cancer progression, metastasis, and treatment response has gained increasing attention. Anticancer treatments (e.g., RT) impact the TME and therefore may alter tumor progression and response to treatments (3–5). Leroi et al. previously demonstrated the impact of the RT schedule and timing of surgery on tumor dissemination in a neoadjuvant RT (NeoRT) model (RT applied before surgery) (6). Indeed, mice operated on earlier after hypofractionated RT developed more extensive metastasis than mice operated on later after the completion of RT treatment. Interestingly, these results are in line with clinical observations that patients operated on within 5 days after RT have worse overall survival (OS) and disease-free survival (DFS) rates than patients who received curative surgery after a treatment-free window of more than 5 days (7). No difference in local control was observed between the two patient groups. The effect on the OS rate is mostly due to a reduction in distant control and a higher tendency to develop metastases. In their study, Leroi et al. demonstrated that the TME was modified after NeoRT and apparently promoted tumor cell dissemination at the time of the surgical procedure. These TME changes depend on the RT schedule applied and seem to be a dynamic process evolving over time, underlying the importance of the timing of the surgery after NeoRT.

From the perspective of clinical application, the neoadjuvant approach has clearly proven its benefits, but the surgical timing has always been a topic of debate (7). The time between the end of NeoRT and surgery allows, on the one hand, for a reduction in the tumor size for R0 resection and improved local control but, on the other hand, allows time for the dissemination of tumor cells. Moreover, as RT modifies the TME and possibly tumor dissemination, a clinical tool for recording such TME modifications is clearly necessary for improving anticancer treatments (8, 9).

Functional imaging is of increasing importance in the field of oncology. It offers the opportunity to noninvasively study several biological processes (10–12). Functional imaging can also predict tumor response earlier than conventional medical imaging and thus may impact treatment strategies (13, 14). Magnetic Resonance Imaging (MRI) is a non-irradiation-based technology offering the advantage of combining conventional anatomic imaging and functional imaging in one setting.

Moreover, an important panel of different functional imaging techniques has already been proven useful in several cancers (15, 16). Diffusion-Weighted MRI (DW-MRI) provides information about water motion and allows tissue characterization (differentiation between tumor and benign tissue), monitoring of treatment response, early detection of recurrent disease, or differentiation between a persistent tumor and a post therapeutic tumor change (17). One specific application of DW-MRI is the Intra Voxel Incoherent Motion (IVIM) theory (18, 19). IVIM theory is based on the differences in the speed of the displacement of water molecules between the tissue and blood vessels. Based on this approach, a variety of information can be extracted, such as pure molecular diffusion parameters (D) and perfusion-related diffusion parameters (D*, f). The results of several studies have demonstrated the value of IVIM analysis in oncology, particularly to characterize molecular prognostic factors (20–24). Dynamic contrast enhancement (DCE-MRI) provides noninvasive, objective assessment of blood flow, tumor perfusion, vessel permeability, and oncotic pressure, and can even predict patient response to anticancer treatments (11, 25–30).

In this study, we used MRI IVIM sequences, and DCE-MRI to characterize the tumor microenvironment modifications induced by NeoRT. Our findings advance the hypothesis that NeoRT induces tumor microenvironment modifications, and that these changes are dynamic over the time period between the completion of RT and surgery. Thus, MR imaging could be used to track these time-dependent modifications over time to assess the optimal window for surgery for an individual patient.

## Material and Methods

### Cell Culture

Human breast cancer MDA-MB-231 cells were used as previously described (31). Cells were grown in Dulbecco’s Modified Eagle’s Medium (DMEM) supplemented with 10% Foetal Bovine Serum, L-glutamine (2 mM) and penicillin (100U/ml)-streptomycin (100 µg/ml). All culture reagents were purchased from Gibco-Life Technologies (Invitrogen Corporation, Paisley, Scotland). For tumor-bearing mice model, the cells were trypsinized and resuspended in serum-free DMEM (1x10^6^ cells/200 µl).

### *In Vivo* Tumor-Bearing Mice and Model

Female 6-8 weeks old SCID mice (Janvier lab, France) were used for all experiments. All animals were maintained at the “Animalerie Centrale” of the University of Liège in a confined area and were monitored daily for all experiments. A mixture (400 µl) of homemade Matrigel (32) and tumor cell suspension (1:1) was injected subcutaneously in the flank of SCID mice as previously described (31). When the tumor volume reached 400 mm³ (which was obtained around 5 weeks after injection), mice were randomly assigned in the different treatment groups. All the experiments were performed in accordance with the ethical committee of the University of Liège (Ethical Protocol 1491). Mouse cohort was n=11 for control group, n=16 for IVIM follow up.

### Radiotherapy and Surgery Protocol

Tumor-bearing mice were locally irradiated with a dose of 10 Gy in 2 fractions administrated in two consecutive days, with a dedicated small animal radiotherapy device (SmART Irradiator from Precision X-Ray Inc, ….). Radiations were delivered using a photon beam (maximum energy of 225 kV and 13 mA), which provided a dose rate of 3 Gy/min. The planning system SmART-plan (version 1.3.9 Precision X-ray, North Branford, CT) was used to establish the *in-vivo* dosimetry and to deliver the treatment. Before irradiation, mice received continuous isoflurane gas anaesthesia *via* induction in an anaesthesia chamber (0, 5 L/min oxygen with 4.0% isoflurane). During irradiation, mice received continuous isoflurane anaesthesia gas *via* a nose cone (0, 4 L/min oxygen with 1.5% isoflurane).

Tumor-bearing mice were operated on the 4th (D4), or on the 11th day (D11), after the end of the RT treatment. This surgery timing and radiotherapy schedule were all derived from a clinical observation and adapted for the preclinical study as previously described (6). Mice were also operated at the time when we observed an increase of perfusion signal (F) on diffusion-weighted MRI. Tumors were carefully removed with a surgical resection including a margin of 3 mm of healthy skin. The skin was sutured with 5-0 silk (Perma-hand, Ethicon). Tumor fragments were formol-fixed and paraffin-embedded or frozen in the air phase of liquid nitrogen for protein and RNA extractions. After surgery, mice were kept alive until D45. At sacrifice, lungs were formol- fixed and paraffin-embedded. To study the functional vascular network, 200 μl of dextran/fluorescein isothiocyanate (FITC) (2.5 mg/ml in PBS) (Sigma-Aldrich, Belgium) was intravenously injected 3 min before surgery as previously described (33).

### MRI and Functional Imaging

MRI was performed on a 9.4-T Agilent VNMRS scanner (Agilent Technologies, Santa Clara, CA, USA) with a volumetric coil (Millipede, Agilent Technologies, Santa Clara, CA, USA). Mice were anesthetized with isoflurane in O_2_ at a concentration of 4% for induction followed by 1–2% for maintenance. Mice were then positioned in the center of the magnet bore, maintained at 37°C with heated cell (Minerve, France), and monitored using respiratory bellows and a rectal temperature probe (SAI Instruments, New York, USA). Mice were also fixed in the cell to avoid movement during acquisition. Imaging was first performed before irradiation and then every day or every two days in case of DCE-MRI, to allow complete washout of the contrast agent between two MRI acquisition. Location sequence, Fast Spin Echo Multi-Slice (FSEMS) axial images were collected (TR = 1500 ms, ESP= 8 ms, Segment/ETL= 96/2, average = 1). For each tumor, we performed 8 consecutive slices of 1 mm with a 0.5 mm gap, a field of view: 30 X 20 mm^2,^ and matrix: 64 X 48. Diffusion-weighted MRI (DW-MRI) was acquired after location sequences, FSEMS axial images (TR = 3000 ms, ESP = 6,21 ms, Segment/ETL= 4/16, average = 2) was performed with 10 b-values (0, 40, 80, 120, 160, 200, 250, 500, 750, 1000) in the three main directions. For dynamic contrast enhancement MRI (DCE-MRI), we carried out a T1 mapping with multiple TR technique in an FSEMS sequence (ESP = 8 ms, Segment/ETL= 96/2, average = 1). For DCE acquisition, FSEMS (TR = 1500 ms, ESP = 8 ms, Segment/ETL= 96/2, average=1) was performed with 200 repetitions of 3s each and Gadobutrol at 0,05 mmol/Kg diluted for a volume of 100 μl (GADOVIST 1,0 mmol/mL, BAYER HEALTHCARE, Loos, FRANCE) IV injection after 10 repetitions.

### MRI Analysis

MRI data were analyzed using in-house-developed programs written in Matlab (R2013a) software (Mathworks, Inc.). For all analyses, a region of interest (ROI) encompassing the tumor was drawn manually on the T2w anatomical image using MRIcron software (34) (www.mricro.com) (see supplemental: **Supplementary Figure S1A**). For DW-MRI, we applied intravoxel incoherent motion (IVIM) (18) analysis using a home-made routine and SPM12 (http://www.fil.ion.ucl.ac.uk/spm/software/spm12/), running in (R2013a) software (Mathworks, Inc.). We applied a denoising filter by subtracting the value of individual noise imaging to all the analyzed images. For each mouse and each b-Value image, diffusion was analyzed voxel per voxel in each direction (X, Y, and Z). Mono-exponential defined by SI/SI0 =exp(–bADC), as well as bi-exponential fitting defined by SI/SI0 =(1–f)·exp(–bD)+ f·exp(–bD∗), where SI0 is the mean signal intensity of the region of interest (ROI) for a b value of 0, and SI is the signal intensity for higher b values, were done voxel-by-voxel to obtain the ADC and IVIM (D, D*, and F) maps, respectively. Voxels with a negative F value were excluded and an averaged F value over the whole tumor was computed (see supplemental: **Supplementary Figure S1B**). Moreover, the distribution of voxels (expressed in percentage of the total voxel in the tumor) based on their F value was calculated. This allows to illustrate the nature of the spatial distribution of F (local or global increase of the perfusion).

For DCE-MRI, we applied semi-quantitative analysis using a home-made routine in Matlab (R2013a) software (Mathworks, Inc.). Briefly, a T1 mapping was obtained with VNMRI (Agilent Technologies, Santa Clara, CA, USA). This T1 mapping is based on multiple TR technique, obtained with a Fast Spin Echo sequence. Then, in Matlab, T1 mapping was subtracted from all DCE images to get images correlated with the contrast injection. For each voxel, we generated a curve of contrast modifications, and we excluded all voxels for which the curve was not compatible with a contrast injection curve with the following criteria: an increase of the signal during the first 15 frames followed by a stability or a decrease of the value (see supplemental: **Supplementary Figure S1C**). After that, we averaged all the data, and the semi-quantitative model-based parameters were calculated automatically. These parameters included AUC (area under the dynamic curve after different timing), Peak Value (maximum contrast enhancement), WashinSlope (Peak Value/time to peak) and Wahsoutslope [(final Signal -Peak Value)/(total time – time to peak)].

### Immunohistochemistry (IHC)

Slides (5 µm thick) were autoclaved in Target Retrieval Solution (Dako, S1699, Denmark), incubated in Proteinase K (S3004, Dako, Denmark) or with EDTA- buffer (Prosan, Belgium) according to the immunolabelling for Ki67, CD31, and FITC-dextran, respectively. Endogenous peroxidases were blocked by 3% H2O2/H2O (Merck, Belgium) for 20 minutes, and nonspecific binding was prevented by incubation in PBS/Bovine Serum Albumin 10% (Fraction V, Acros Organics, NJ). Tumor sections were incubated with a mouse monoclonal anti-human Ki-67 antibody (1/100) (clone MIB-1, M7240; DAKO, Denmark), a rat anti-CD31 antibody (1/100) (Ab56299, Abcam, United Kingdom), or a ready-to-use anti-fluorescein antibody (Converter-POD, Roche, France). After 3 washes in PBS or Tris-HCl for CD-31 staining, slides were incubated with an HRP-conjugated secondary antibody, after post antibody blocking (DPVB Blocking, Immunologic NL) for pimonidazole staining, and revealed with Vector DAB (SK-4100, Vector Laboratories, Burlingame, CA, USA). Slides were counterstained with hematoxylin. For lung metastases quantification, six lung sections of 5 µm, spaced by 10 sections of 5 µm, were immunostained with an antibody against human Ki67 as previously described (6). Metastases were manually counted and classified according to their size (<10 cells, 10 to 50 cells, 50 to 100 cells, >100 cells).

### Histological Image Processing and Computerized Quantification

Immunostained sections were scanned using the digital slide scanner NanoZoomer 2.0-HT system at 0.46 μm/pixel (20X) scanning resolution (Hamamatsu, Mont- Saint-Guibert, Belgium) and images were registered in the RGB (red, green, blue) color space. Necrotic and stromal areas were eliminated using the hybrid human-computer approach described previously (35). For blood vessel and functional blood vessels quantification within tumor regions of interest, image processing and measurements were performed using the toolbox of image analysis of the MATLAB (R2013a) software (Mathworks, Inc.) according to the algorithms as previously described elsewhere (36). Importantly, binary images resulting from the image processing were systematically compared visually with the corresponding original images and occasionally, the threshold was adapted manually if necessary. The results are expressed as density defined as the measured area occupied by vessels divided by the total area of the corresponding tumor regions of interest or for perfusion as the area of perfused vessels divided by the total area of vessels.

### Statistical Analysis

Statistical analyses were carried out using the Prism 5.0 software (GraphPad, San Diego, CA) and we performed unpaired t-test or Mann- Withney test and ANOVA followed by Bonferonni post-test when requested or Kruskal-Wallis test followed by Dunn’s test when requested. Results were considered significant for p < 0.05 and expressed as means ± standard error of the mean (SEM). *: p < 0.05; **: p < 0.01; ***: p < 0.001; ****: p < 0.0001. ns = non statistically significant.

## Results

### Impact of NeoRT on Tumor Components Recorded by IVIM and DCE-MRI

**Figure 1:**
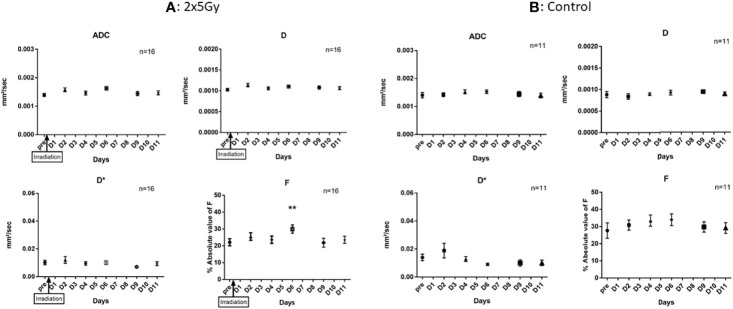
Impact of RT on IVIM parameters. **(A)** In irradiated mice, we observed a significant increase of F parameters at D6 (p < 0,03), we didn’t observe modification of other IVIM parameters. **(B)** In the control group, we didn’t observe any modification of diffusion parameters for 11 days. Results are expressed as mean + SEM. **p < 0.01.

**Figure 2:**
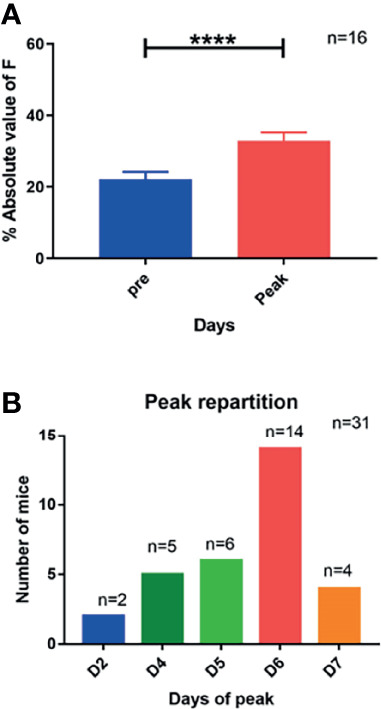
Increase of F parameter after irradiation and time distribution of this increase. **(A)** An increase of nearly 50% of F parameters during the peak is illustrated (mean pre = 23,4; mean peak = 34). **(B)** The distribution of the peak of F parameters is mostly observed at day 6 but shows a distribution around this timing. Results are expressed as mean + SEM. ****p < 0.0001.

Initially, we ensured the possibility of a longitudinal follow-up with MRI (i.e., IVIM) of tumor-bearing mice after the NeoRT treatment. Sham-irradiated mice received the same imaging. The IVIM acquisition consisted of DW acquisition with ADC, D, D*, and F parameters. All groups tolerated the treatment well, without any sign of weight loss or other distress (see supplemental: **Supplementary Figure S2**). During all the procedures, the size of the tumors was recorded and did not significantly differed from the irradiated groups (see supplemental: **Supplementary Figure S2**). The control sham-irradiated mice did not display any modification in any of the diffusion parameters evaluated compared to the basal value. The irradiated group showed a significant increase in the F parameter (n=16, p<0.003), which was related to perfusion, on day 6 after the end of irradiation. No other diffusion parameters (ADC, D, or D*) changed significantly (see **Figure 1**). We reproduced similar results in a syngeneic tumor model but this time, we observed that the F peak occurred earlier at D3 and no modification were observed in the control group (see supplemental: **Supplementary Figure S3**). All irradiated mice had an increase in the F parameter, although this increase occurred at different times after RT. Nearly half of the mice presented the F peak at D6 and 77% from D5 to D7. The F parameter increased by 147% during the peak compared to the pre-RT basal value (mean = 23.44 ± 2.37 before irradiation versus mean= 34.03 ± 2.53 during the peak, n=17). (see **Figure 2**). Of note, the increase was transient and returned to the basal level within 24 hours.

**Figure 3:**
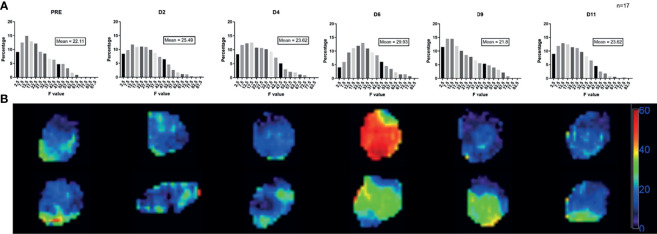
Evolution of F value voxel distribution after irradiation. **(A)** These graphs showed the evolution of the voxel distribution of F value after irradiation. For each day, we expressed the percentage of voxel with a specific value of F. At D6, we observed a shift of voxel distribution with an increase of all range of F value. For the analysis the percentage of voxel repartition per value were done for each tumor than pool together to avoid bias from huge tumor. **(B)** Evolution of F voxel mapping in two different tumors after irradiation, representation at D0, D2, D4, D6, D9 and D11. The scale represents the absolute value of F parameter.

By quantifying the distribution of F value voxel by voxel in the tumor, we observed a global increase in all the tumors at D6 after irradiation (see **Figure 3**). We analyzed the deviation of the voxel distribution compared to the mean value, and we did not observe an increase of hot spots explaining the increase of F value at D6; instead, we observed a global shift to higher value at D6.

**Figure 4:**
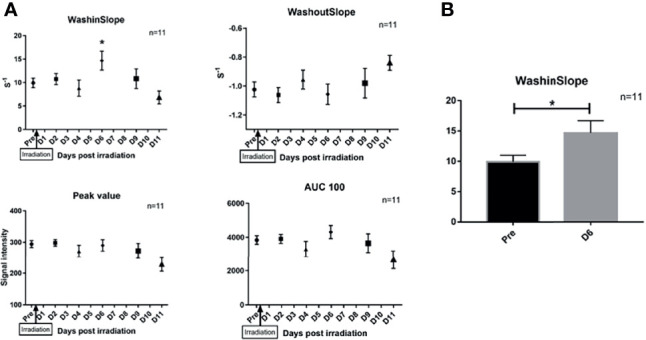
Impact of RT on DCE parameters after irradiation. **(A)** We observed a significant increase of the wash-in-slope at day 6 after irradiation (p < 0,03), we didn't observe modification of other DCE parameters. **(B)** show the evolution of washinSlope before irradiation and at D6 after irradiation (p < 0,03). Results are expressed as mean + SEM. *p < 0.05.

According to the IVIM theory, the F parameter is related to vessel perfusion. Therefore, we used DCE-MRI to confirm the transient increase in perfusion observed after RT. DCE-MRI is appropriate for studying tumor perfusion due to the recording of the washing in and washing out of contrast agent. IVIM and DCE-MRI were acquired one after the other with the same FOV to ensure a perfect geometrical correlation between the images. As previously observed with IVIM, the irradiated mice showed a transient and significant increase in the wash-in slope (p<0.03) at D6 post RT. This wash-in increase of 148% (mean = 9.9 ± 1.1 before irradiation versus mean= 14.67 ± 2.01 during the peak, n=11) was of the same magnitude of change as that observed for the F peak (147%) (see **Figure 4**). The other DCE-MRI parameters, such as the time to peak, wash-out slope, and area under the curve (AUC), at multiple timings were not significantly modified.

### NeoRT Induced Transient Vessel Perfusion

**Figure 5:**
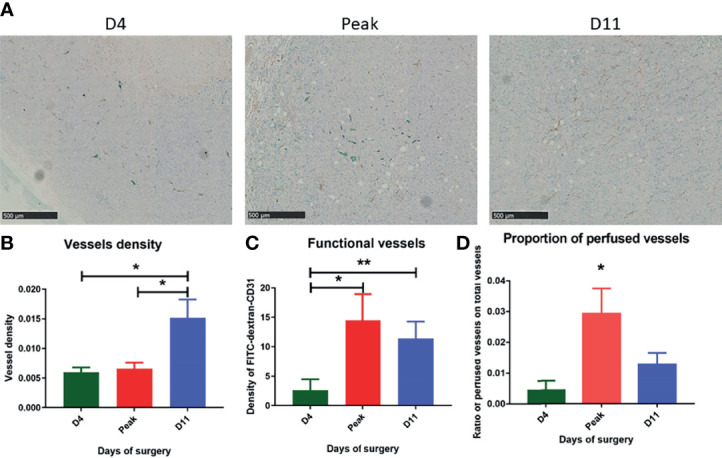
Immunohistochemical analysis performed on tumor depending on the day of surgery. **(A)** Immunostaining with CD31 in brown and FITC dextran in green at different timing of surgery. **(B)** CD31 staining show mature vessel in the tumor, we observe a significant difference between D4, the peak and D11 (mean +/- SEM: D4 = 0.0059 +/- 0.0008 VS Peak= 0.0066 +/- 0.0010 VS D11 = 0.0151 +/- 0.0031). **(C)** Colocalization of FITC and CD31 staining showing functional vessels reveals an increase of functional vessels when surgery is performed during the peak or at D11 compared to D4 (D4 = 2,61+/- 1,86 VS Peak=14,47 +/- 4,45 VS D11 = 11,41 +/- 2,86). **(D)** We quantify the proportion of functional vessel in the tumor and show a significant increase during the peak of F parameter (mean +/- SEM: D4 = 0.0044 +/- 0.0030 VS Peak= 0.0294 +/- 0.0082 VS D11 = 0.0129 +/- 0.0037). Results are expressed as mean + SEM. *p < 0.05. **p < 0.01.

DCE-MRI and DW-MRI (IVIM) demonstrated a transient increase in tumor perfusion, mostly at approximately day 6 after NeoRT. To determine whether there is a correlation between the imaging parameters concerning perfusion and a transient increase in tumor perfusion after NeoRT, we quantified tumor vessel density with CD31 staining and perfused vessels with FITC dextran injected 3 minutes before the surgery. The timing of the surgery and FITC-dextran injection was guided by the peak of F measured by DW-MRI over time, and finally, two additional time points (D4 and D11) were selected according to previously published data (6). The histological analysis demonstrated a significant increase in vessel density at D11 compared to at D4 and at the F peak (mean +/- SEM: D4 = 0.0059 +/- 0.0008 VS Peak= 0.0066 +/- 0.0010 VS D11 = 0.0151 +/- 0.0031) (see **Figures 5A, B**). The assessment of functional vessels by quantitative colocalization between FITC dextran and CD31 revealed an increase in functional vessels when the surgery was performed during the F peak or at D11 compared to when it was performed at D4 (D4 = 2,61+/- 1,86 *vs* peak=14,47 +/- 4,45 *vs* D11 = 11,41 +/- 2,86) (see **Figures 5A, C**). Furthermore, the proportion of perfused vessels in the tumor was determined by the ratio of FITC-dextran-labeled vessels to the total number of CD31-positive vessels. This proportion of perfused vessels was 2-3-fold higher in mice operated on at the F peak than in the other experimental groups (mean +/- SEM: D4 = 0.0044 +/- 0.0030 VS Peak= 0.0294 +/- 0.0082 VS D11 = 0.0129 +/- 0.0037) (see **Figures 5A, D**).

### Surgery Timing Guided by IVIM for Metastatic Spreading

**Figure 6:**
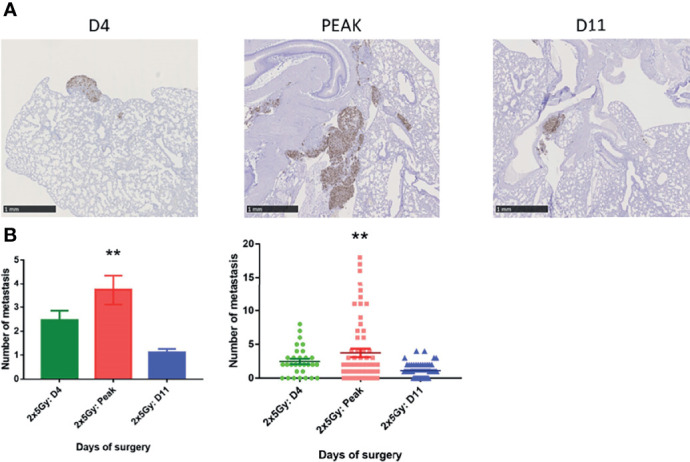
Lung metastasis quantification depending of the day of surgery. **(A)** Representative sections of lungs collected at the end of the experiment. Metastatic cells were labeled with an anti-human Ki67 antibody. **(B)** Average number of global lung metastases (D4 = 2,46+/- 0,38 VS Peak=3,73 +/- 0,62 VS D11 = 1,11 +/- 0,14) Results are expressed as mean + SEM. *p < 0.05. **p < 0.01.

Using MRI, we identified a net transient increase in tumor perfusion when the F parameter peaked. To verify whether this transient increase could impact the metastatic dissemination of the tumor when surgery was performed at the F peak time point, we carefully followed tumor-bearing mice after NeoRT with DW-MRI. When the peak occurred, the tumor was immediately surgically removed. Mice without a peak at D4 or D11 post-NeoRT were used as controls and operated on at these time points (6). Mice were sacrificed 45 days after NeoRT, and lungs were collected for immunohistochemistry analysis. In line with previous results, fewer metastases were detected when surgery was performed at D11 than when it was performed at D4 (34). When surgery was performed at MRI-detected F peak time, the burden of lung metastases was significantly (p<0.01) greater than that in the other experimental groups (number of metastases D4 = 2.46+/- 0.38 VS Peak=3.73 +/- 0.62 VS D11 = 1.11 +/- 0.14) (see **Figure 6**).

## Discussion

In this study, we monitored a previously used neoadjuvant radiotherapy mouse model with MRI to demonstrate the impact of surgical timing on metastasis dissemination (6). It is well known that neoadjuvant treatment has a major impact on the treatment of cancer with a decrease in local recurrence and also has an effect on tumor size, allowing for a possible organ-sparing approach. As a result of neoadjuvant treatment, including RT, patients’ overall survival times are no longer determined by local tumor control, but by the occurrence of distant metastases.

First, we followed tumor modifications with IVIM DW-MRI, and we observed a significant and transient increase in the F parameter 6 days after irradiation. This parameter is linked with perfusion and, specifically, blood volume (37). This link has already been brought to light in some studies in other fields. Alison et al. (38) suggested a direct correlation between an F parameter decrease and placental hypoperfusion, and they also reported a decrease in the F parameter during hyperoxygenation, which is known to induce a decrease in perfusion in tissue. With DCE-MRI, we confirmed this increase in perfusion with an increase in the wash-in slope at the same time as the F parameter, which also correlated with blood flow and tumor vessel permeability. Some studies have already shown the potential value of DCE-MRI for studying parameters longitudinally, such as for monitoring the effects of antiangiogenic treatments (39, 40). Aiming to further confirm these findings, we performed FITC dextran labeling of tissue sections, and we showed that an increase in the perfusion parameter was associated with an increase in the proportion of perfused vessels in the tumor. These results are consistent with those of PAN et al. (41), who demonstrated a correlation between IVIM perfusion parameters and relative perfusion (lectin+ area/CD31+ area).

Previously, Leroi et al. demonstrated the impact of surgical timing on metastatic spread. In their study, they showed that the burden of lung metastases was reduced when surgery was performed at a later time point after RT (6). In our study, by performing surgery guided by imaging, during the peak of the F parameter, we observed an increase in metastasis burden. We provide evidence that the increase in the MRI-based F parameter indicates a switch in blood vessel perfusion associated with increased metastatic dissemination. This is also in line with a clinical study that revealed a correlation between an increase in relative perfusion in the tumor and the presence of metastases in patients with rectal cancer (42). A possible explanation for the connection between the increase in perfusion and metastasis dissemination could be the presence of circulating tumor cells (CTCs). There is a link that has been described in some publications between tumor “manipulation” during surgery and the subsequent appearance of CTCs in liquid biopsies (43).

MRI is a powerful, noninvasive imaging tool that can be used to assess many aspects of basic tissue functions and parameters, such as tumor perfusion, which can be quantified by DCE-MRI (44). Moreover, DW-MRI is an interesting sequence for characterizing the stromal microenvironment, which is associated with progression, metastasis, and, hence, prognosis (45). To the best of our knowledge, a longitudinal approach in the objective assessment of tumor modifications to predict metastatic spread after neoadjuvant RT with a noninvasive imaging method has never been described before. This original approach allowed us to evaluate the evolution over time of vessel features and to identify a measurable and reliable parameter that indicates a key modification in the tumor environment that could predict metastatic dissemination. We identified the first MRI-determined parameter associated with vessel perfusion that could guide the timing of tumor surgery after radiotherapy.

There are some limitations to our study. First of all, further studies are requested both *in vivo* as well as in humans, to confirm our observation of a correlation between F parameter, increase in vessel perfusion and appearance of metastasis. Moreover, while we limited our approach to the use of gadolinium, to estimate DCE-MRI, we are well aware that other contrast agents might be better suited for the characterization of vascular changes and interstitial pressure within the tumor (46). One could argue that a semi quantitative DCE-MRI analysis is a simplistic solution to estimate tumor perfusion, as compared to a well-established model such as Tofts model. This latter yields a quantitative estimation and offers a better understanding of the exchange between different compartments (47). However, we need therefore the arterial input function (AIF) for normalization of tumor changes according to the evaluation of contrast concentration over time within the blood circulation. In order to have AIF and tumor perfusion simultaneously, one should consider modifying our field of view, compared to the one used for IVIM acquisition. We decided to keep the conditions for DW-MRI and DCE-MRI identical (identical FOV, number and localization of slices) and therefore performed only a semiquantitative analysis. We intended mainly to highlight a change in perfusion with DCE-MRI. We are well aware that doing so, information is lost (vessel permeability, volume in the extravascular space, and various quantitative parameters) (47). A third limitation is the use of an immunodeficient mouse model, these limitations have been discussed elsewhere in detail (6). But in a syngeneic model, similar modifications of IVIM have been observed. In such a syngeneic model, we were confronted with rapid tumor growth as well as metastatic dissemination. Indeed, the transient increase of F occurred at day 3 instead of day 6, and metastases appeared rapidly as bulky mediastinal masses. Therefore, the immunodeficient model is more adequate and more representative for a clinical context. However, it is obvious that a humanized mice model, would better explain the interplay between neoadjuvant radiotherapy and surgery (48). Without any doubt many biological mechanisms need to be explored to fully understand the interaction of with treatment modalities and transient changes in tumor perfusion (7).

Locally advanced Rectal tumor are actually treated with hypofractionated (5x5Gy) neoadjuvant radiotherapy and the best timing for surgery is still on debate (49). Several publications described the usefulness of DW-MRI and ADC to predict tumor response, but none of them evaluated the tumor perfusion profile and its modifications over time after radiotherapy (50, 51). Our work demonstrated the interest of such a follow up. Nevertheless, repeated MRI for studying radiotherapy induced tumor modifications over time in human, might be difficult to envision due to the limited access to MRI and the consent of the patient for a daily examination. However, IVIM-MRI avoids contrast injection making it easier for the patient to accept repeated examination. The introduction in the clinical routine of the MR-Linac (e.g. Elekta Unity^®^), a machine combining a MRI and a radiotherapy machine may provide a solution. This machine allows to acquire IVIM and DCE-MRI in the same time as treatment delivery, offering the opportunity to study very early tumor modifications induced by the treatment (52).

In conclusion, our study provides new perspectives to the field of MRI and oncology. We have demonstrated the ability to monitor the impact of radiotherapy on the tumor microenvironment in a noninvasive manner. We showed a close correlation between imaging features and biological characteristics during neoadjuvant treatment. DW-MRI and DCE-MRI approaches allowed us to identify when the risk of metastatic dissemination was the greatest after NeoRT. This emphasizes the importance of surgical timing after NeoRT, which is still a subject of huge debate in the clinic. It is worth noting that both MRI modalities are routinely available in the clinic, thus facilitating the translation of our results to patients. Bringing us one step closer to an individualized approach, our methods could be used to help develop personalized treatments and guarantee the best patient outcome possible.

## Data Availability Statement

The raw data supporting the conclusions of this article will be made available by the authors, without undue reservation.

## Ethics Statement

The animal study was reviewed and approved by Ethical committee of the University of Liège (Ethical Protocol 1491).

## Author Contributions

FL, NL, AP, and PM contributed to conception and design of the study. FL and NL performed the experience and analysis. FL wrote the first draft of the manuscript. All authors contributed to manuscript revision, read, and approved the submitted version.

## Funding

This work was financially supported by Fonds National pour la Recherche Scientifique (FNRS, Télévie Grant n°17032258), Fonds pour la Recherche Scientifique et Médicale (FRSM, Grant n°19491986) and the Fonds Léon Fredericq.

## Conflict of Interest

The authors declare that the research was conducted in the absence of any commercial or financial relationships that could be construed as a potential conflict of interest.

## Publisher’s Note

All claims expressed in this article are solely those of the authors and do not necessarily represent those of their affiliated organizations, or those of the publisher, the editors and the reviewers. Any product that may be evaluated in this article, or claim that may be made by its manufacturer, is not guaranteed or endorsed by the publisher.

## Supplementary Material

Supplementary Figure S1 Mask, IVIM and DCE quantification. **(A)** Manual creation of the mask on a T2 anatomical sequence. **(B)** IVIM and ADC analysis on the tumor previously map. **(C)** Semi-quantitative DCE analysis on the same tumor.

Supplementary Figure S2 Tumor volume at surgery and evolution of mice weight.

Supplementary Figure S3
**Figure 1** Impact of RT on IVIM parameters in a syngeneic mice model. In this syngeneic model, 200 000 4T1 cells are injected in the flank of BalbC mice. When the tumor reached 100 mm^3^ generally after 1 week, we irradiated the tumor with 2x5Gy as previously described. Mice were following by DW MRI with the same MRI protocol as described in the M&M. MRI measurement performed every day or every other day after irradiation for 7 days to record tumor modification after irradiation. In this rapid growing tumor model, the increase of F parameter is observed earlier (D3) than in Scid mice, the rapidity of the tumor growth in this model can explain this difference of timing. **(A)** In irradiated mice, we observed a significant increase of F parameters at D3 (p < 0,03), we didn’t observe modification of other IVIM parameters. **(B)** In the control group, we didn’t observe any modification of diffusion parameters for 7 days. Results are expressed as mean + SEM. *p < 0.05.
