# Supplementary figures and images for: Tumor Microenvironment Modifications Recorded With IVIM Perfusion Analysis and DCE-MRI After Neoadjuvant Radiotherapy: A Preclinical Study
Source: Front Oncol. 2021 Dec 21;11:784437. doi: 10.3389/fonc.2021.784437 (PMC8724034; doi:10.3389/fonc.2021.784437)

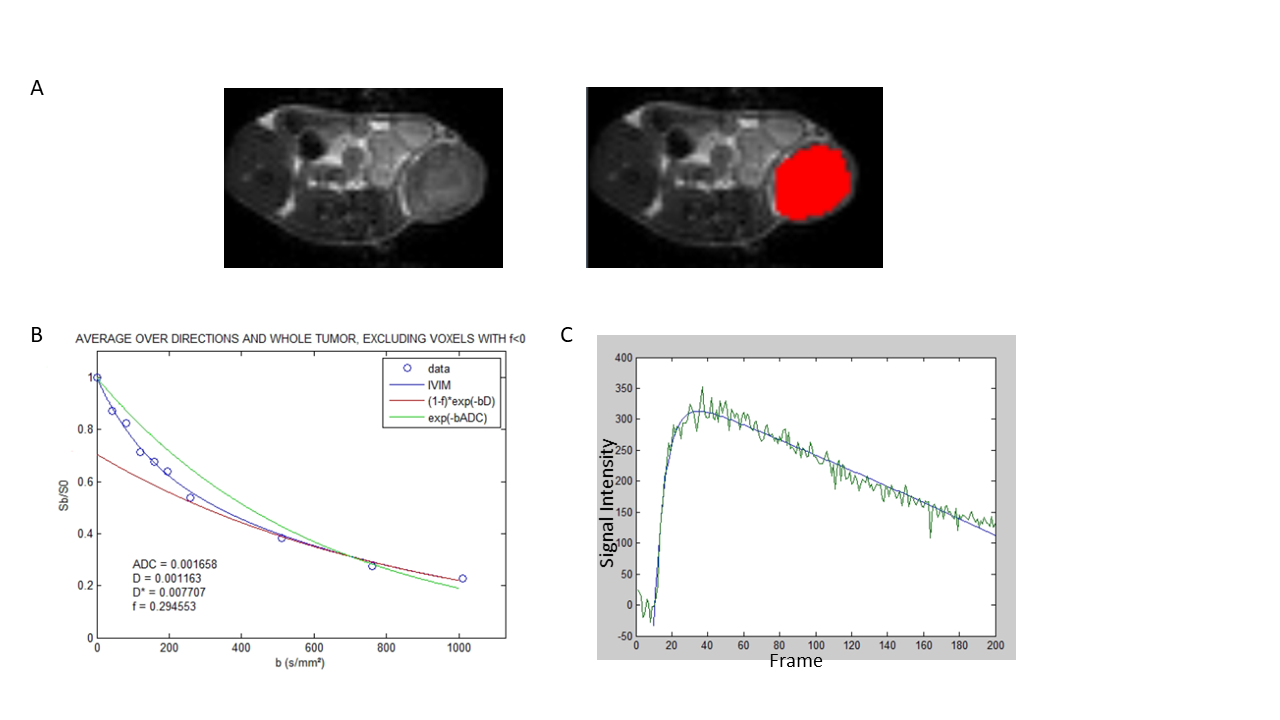

Supplement: Supplementary Figure S1 — Mask, IVIM and DCE quantification. (A) Manual creation of the mask on a T2 anatomical sequence. (B) IVIM and ADC analysis on the tumor previously map. (C) Semi-quantitative DCE analysis on the same tumor. [file Image_1.tif]

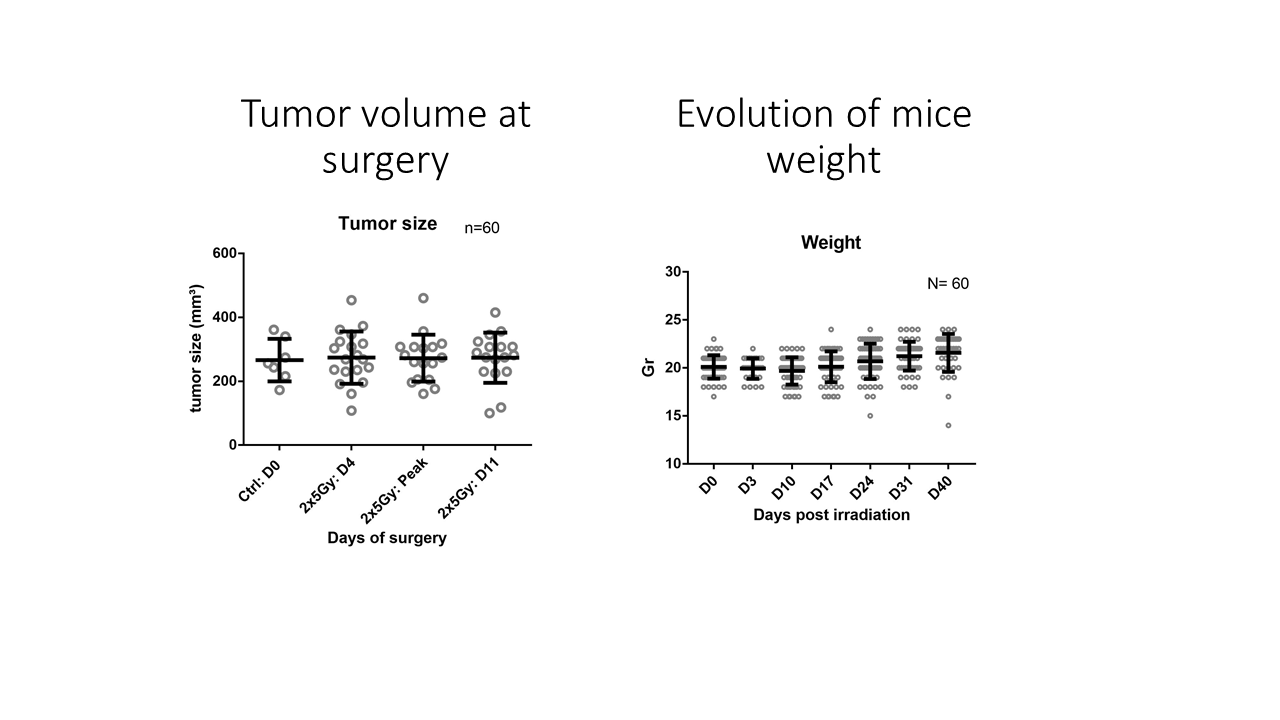

Supplement: Supplementary Figure S2 — Tumor volume at surgery and evolution of mice weight. [file Image_2.tif]

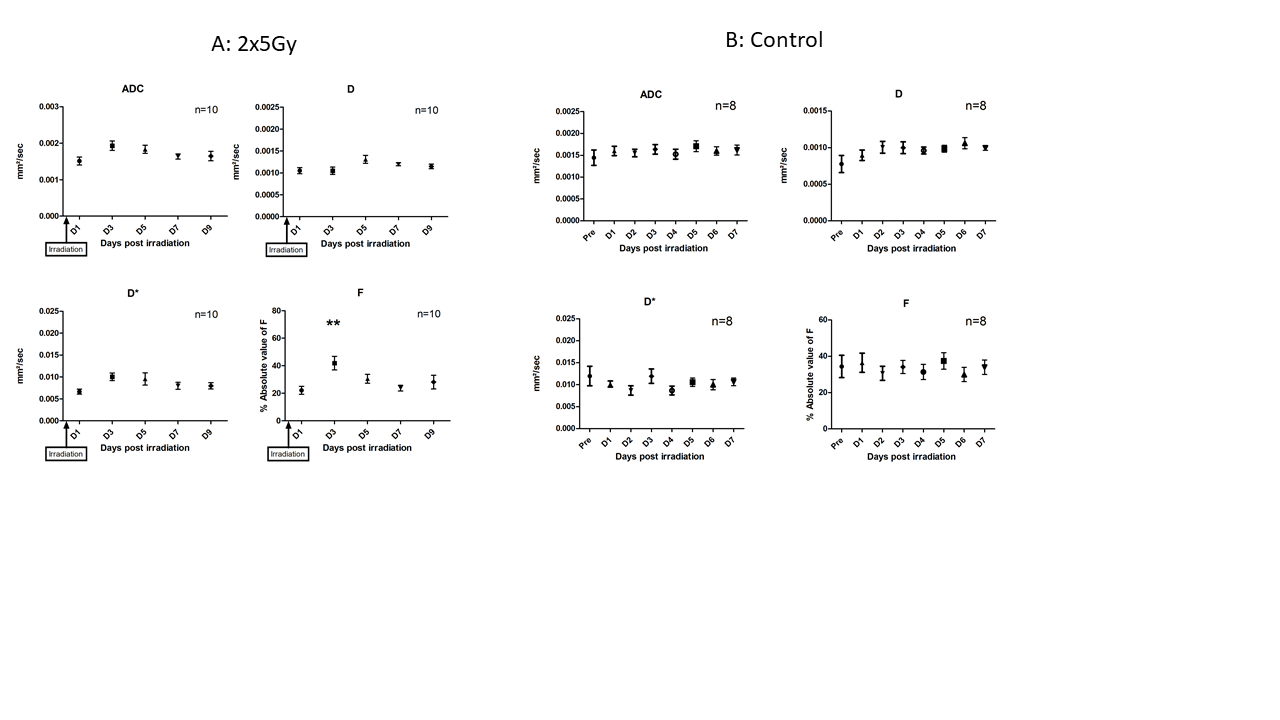

Supplement: Supplementary Figure S3 — Figure 1 Impact of RT on IVIM parameters in a syngeneic mice model. In this syngeneic model, 200 000 4T1 cells are injected in the flank of BalbC mice. When the tumor reached 100 mm3 generally after 1 week, we irradiated the tumor with 2x5Gy as previously described. Mice were following by DW MRI with the same MRI protocol as described in the M&M. MRI measurement performed every day or every other day after irradiation for 7 days to record tumor modification after irradiation. In this rapid growing tumor model, the increase of F parameter is observed earlier (D3) than in Scid mice, the rapidity of the tumor growth in this model can explain this difference of timing. (A) In irradiated mice, we observed a significant increase of F parameters at D3 (p < 0,03), we didn’t observe modification of other IVIM parameters. (B) In the control group, we didn’t observe any modification of diffusion parameters for 7 days. Results are expressed as mean + SEM. *p < 0.05. [file Image_3.tif]
